# Probing Protein Structure and Folding in the Gas Phase by Electron Capture Dissociation

**DOI:** 10.1007/s13361-015-1088-z

**Published:** 2015-04-14

**Authors:** Moritz Schennach, Kathrin Breuker

**Affiliations:** Institute of Organic Chemistry and Center for Molecular Biosciences Innsbruck (CMBI), University of Innsbruck, Innrain 80/82, 6020 Innsbruck, Austria

**Keywords:** Protein structure, Folding, Gas phase, Electron capture dissociation, Mass spectrometry

## Abstract

The established methods for the study of atom-detailed protein structure in the condensed phases, X-ray crystallography and nuclear magnetic resonance spectroscopy, have recently been complemented by new techniques by which nearly or fully desolvated protein structures are probed in gas-phase experiments. Electron capture dissociation (ECD) is unique among these as it provides residue-specific, although indirect, structural information. In this Critical Insight article, we discuss the development of ECD for the structural probing of gaseous protein ions, its potential, and limitations.

**Graphical Abstract:**
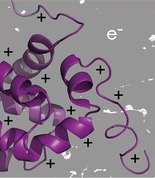
ᅟ

ᅟ

## History

**Scheme 1:**
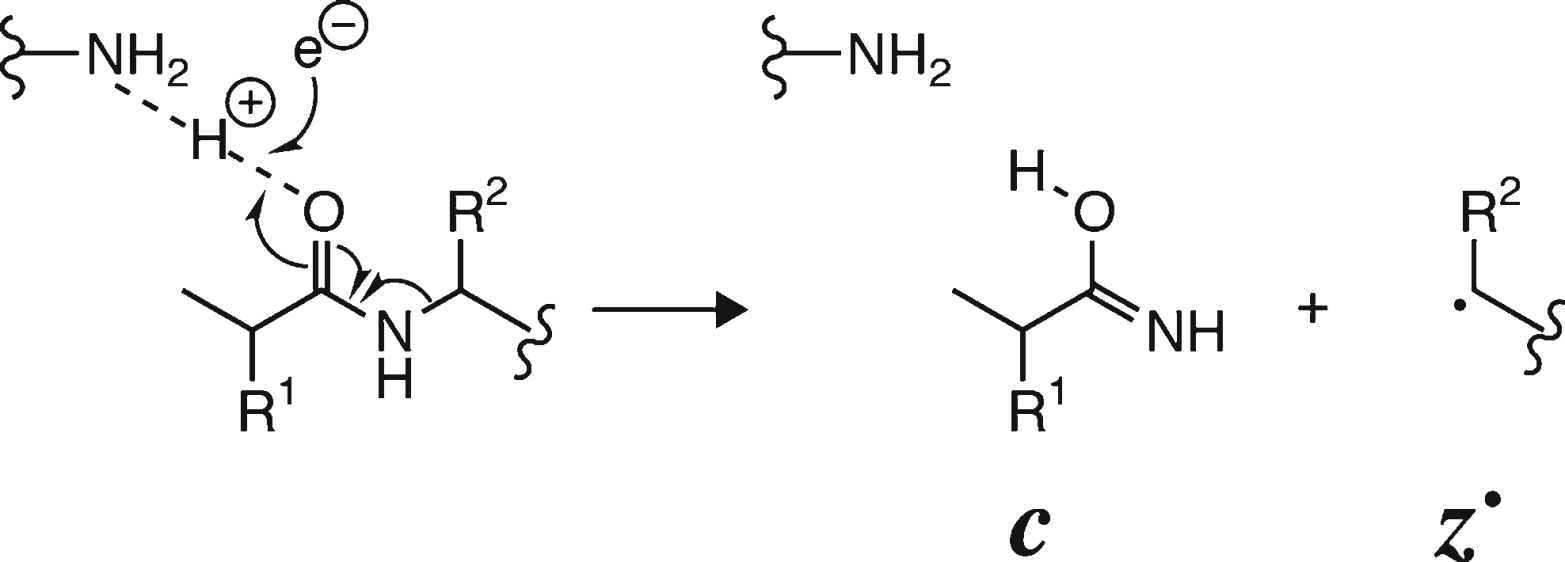
Proposed mechanism for the formation of ***c*** and ***z*** ^•^ fragments by ECD [32]; that for the far less common dissociation into ***a*** ^•^ and ***y*** fragments is not discussed here

Shortly after their breakthrough discovery of electron capture dissociation (ECD) [1], McLafferty and coworkers reported that electron capture by (M + 34H)^34+^ ions of carbonic anhydrase (~29 kDa) [2] and (M + 33H)^33+^ ions of an IgE construct (~49 kDa) [3] gave only signals corresponding to molecular ions with reduced charge [i.e., (M + nH)^(n-1)+•^, (M + nH)^(n-2)+••^, etc.] but no products from dissociation [i.e., ***c*** or ***z*** ^•^ fragments from protein backbone cleavage (Scheme 1)]. This experimental finding was attributed to convoluted higher order structures of the protein ions that prevented separation of the ***c*** and complementary ***z*** ^•^ fragments [2]. In subsequent work [4], vibrational ion activation by energetic collisions with N_2_ gas during exposure to low-energy electrons (“activated ion ECD”) was used to break intramolecular noncovalent bonds in proteins of up to ~42 kDa, which significantly increased the number of ***c*** and ***z*** ^•^ fragments.

Importantly, collisional activation or exposure to infrared (IR) photons of (M + 8H)^7+•^ ions of ubiquitin gave no ***b*** and ***y*** fragments, which are typical products of slow (M + nH)^n+^ ion heating, but instead, ***c*** and ***z*** ^•^ fragments characteristic for ECD, whose site-specific relative abundances were substantially different from those in the ECD spectrum of (M + 8H)^8+^ ions [2]. Vibrational activation of (M + nH)^(n-1)+•^ ions can also yield ***c*** ^•^ and ***z*** fragments, presumably from hydrogen transfer between two complementary ***c*** and ***z*** ^•^ fragments that are still held together by noncovalent bonding [5–7]; ***c*** ^•^ and ***z*** ions generally disappear when the (M + nH)^n+^ ions are unfolded prior to or during reaction with electrons [7, 8]. In yet another study, ~90% dissociation of ubiquitin (M + 7H)^6+•^ ions into ***c*** and ***z*** ^•^ (and ***c*** ^•^ and ***z***) fragments was obtained by vibrational excitation at far lower energies (~10%) than those required for collisionally activated dissociation (CAD) or infrared multiphoton dissociation (IRMPD) of (M + 7H)^7+^ ions into ***b*** and ***y*** fragments, consistent with breaking of noncovalent instead of covalent bonds [9]. This was interpreted as evidence for ECD data reflecting protein structure in the gas phase, in that the observation of separated ***c*** and ***z*** ^•^ fragments indicates the lack of noncovalent bonding between each other [9]. Ever since, the unusual capability of ECD to break covalent N–Cα bonds while maintaining noncovalent bonds that account for the higher order structure of gaseous ions has been used to obtain information about peptide or protein structure in the absence of solvent [7, 9–25]. More recently, this approach has been extended to the study of noncovalently bound protein complexes [18, 26–31].

## Formation of ECD Fragments

**Figure 1:**
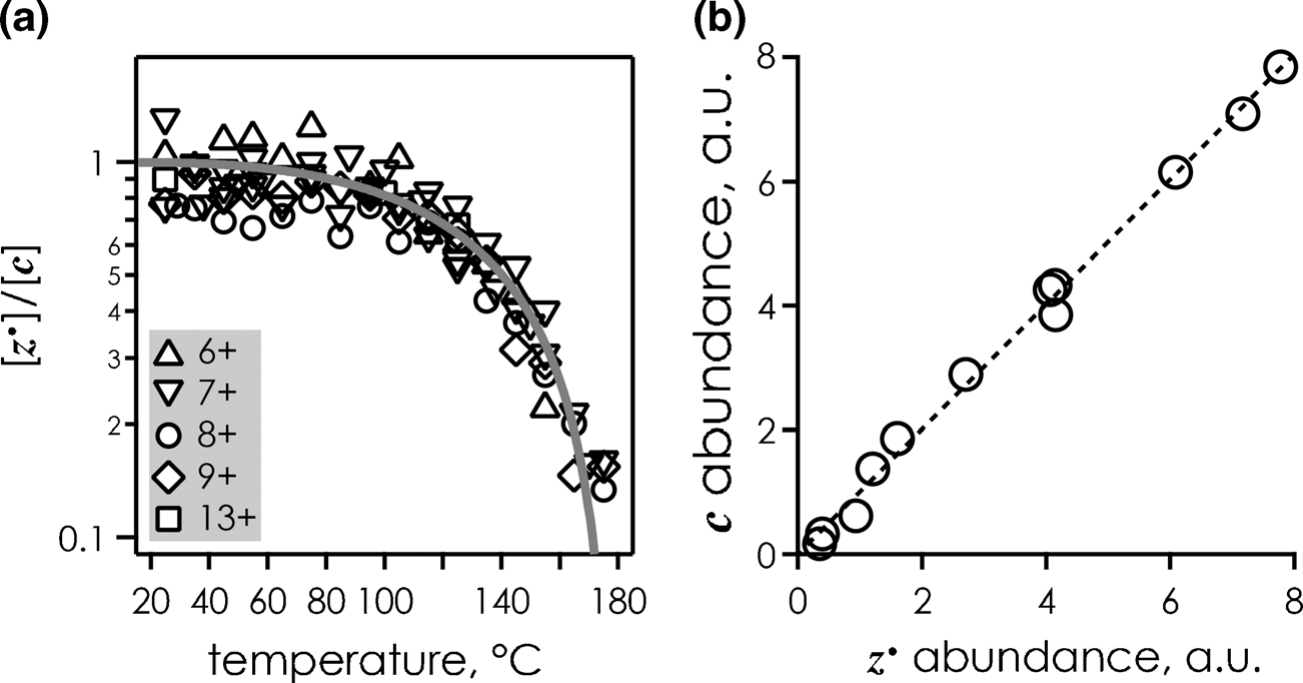
(**a**) Ratio of ***z*** ^•^ and ***c*** fragments from ECD of ubiquitin (M + nH)^n+^ ions thermalized by blackbody infrared irradiation (data from references [9, 12]), the solid line is meant to guide the eye; (**b**) abundances of ***c*** versus ***z*** ^•^ fragments from ECD of KIX (M + nH)^n+^ ions (*n* = 7–16) at room temperature (*circles*, data from reference [19]), *dashed line* with unit slope indicates equal abundances [for *n* = 11 and 12, data from ECD of (M + nH)^n+^ ions from ESI of both “native” and “quasi-native” solutions are included]

The mechanism of protein backbone bond cleavage into ***c*** and complementary ***z*** ^•^ ions is still debated, but it is generally agreed that ECD involves positively charged sites [1, 2, 33–39]. Moreover, it was shown that heating (from 25 to 125°C) of ubiquitin (M + 13H)^13+^ ions, which have an extended structure with a collision cross-section (CCS) of ~2115 Å^2^ at room temperature that is largely unaffected by collisional activation [40], does not appreciably affect the overall or site-specific yields of ***c*** ions from ECD [12]. This means that ECD cleavage is equally facile at ion temperatures of 25, 100, and 125°C, although the thermal stability of the ***c*** and ***z*** ^•^ fragment ions is substantially different (Figure 1a). Consistent with their radical nature, ***z*** ^•^ ions are far less stable than the even-electron ***c*** ions, and spontaneously undergo secondary dissociation at higher ion temperatures, which makes them less useful for structural probing in thermal unfolding experiments [9].

## Excess Energy in ECD

Is it possible that any excess energy in ECD [i.e., the energy from cation-electron charge recombination (CR) that is not consumed by N–Cα bond cleavage] causes secondary dissociation of the radical ***z*** ^•^ ions, or even changes in the higher order structure of the protein ions? At room temperature, ECD of linear proteins generally produces very similar yields of ***c*** and ***z*** ^•^ ions (Figure 1b), which shows that any excess energy must be significantly smaller than that required for secondary dissociation of the radical ***z*** ^•^ ions. For peptides and proteins that comprise cyclic backbone or disulfide bonded structures, secondary radical reactions can, nonetheless, proceed at room temperature [3, 20, 41], most likely driven by the energy released by breaking up the strained cyclic structures.

For energy minimized forms of the ubiquitin crystal structure in the absence of solvent, Williams and coworkers have conservatively estimated that capture of one and two electrons, with assumed CR energies of 6 and 12 eV, is associated with a temperature increase from ~20 to ~85 and ~140°C, respectively [17]. Calculated CR energies for small (~130 to 1115 Da, 18 to 172 atoms) linear peptide ions with *m/z* values between ~100 and 555 and up to three charges did not exceed 6.6 eV, and generally increased with increasing charge [42]. For example, the adiabatic CR energy for (AHDAL +2H)^2+^ ions with *m/z* 264, 5.53 eV, was about twice as high as that for (AHDAL + H)^+^ ions with *m/z* 526, 2.93 eV. Vertical CR energies were even smaller by an average factor of 0.8, in good agreement with the CR energy of 4.3 eV for (KYK + 2H)^2+^ ions at *m/z* 220 determined in nanocalorimetry experiments [43].

**Figure 2:**
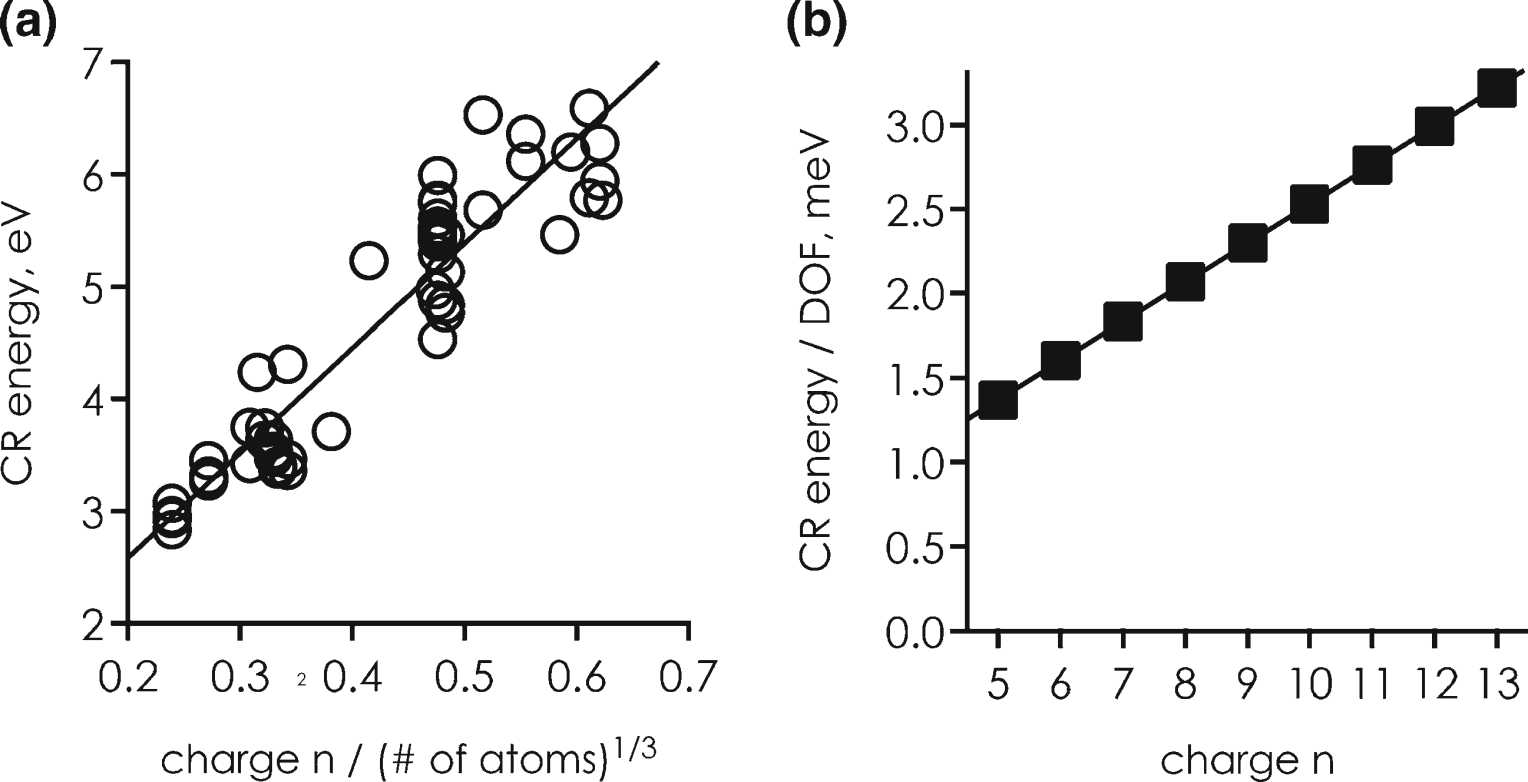
(**a**) Calculated adiabatic CR energies of small peptide ions from reference [42] versus charge divided by the cube root of the ion’s number of atoms, *solid line* shows linear fit with r^2^ = 0.874; (**b**) estimated CR energies for ubiquitin (M + nH)^n+^ ions per vibrational degree of freedom (DOF)

We found that the calculated CR energies increased roughly linearly with peptide ion charge divided by the cube root of the ion’s number of atoms as illustrated in Figure 2a. Extrapolation of the linear fit function to *n* = 0 gives an intercept of 0.721 ± 0.213 eV, which can be interpreted as the average electron affinity (EA) of gaseous peptides with zero net charge. Other neutral compounds with EA values in this range include inorganic salts such as sodium chloride (0.727 eV [44]) and potassium iodide (0.728 eV [44]), whereas small model compounds that more closely resemble the chemical structure of peptides, such as formamide (0.017 eV [44]), N-methylformamide (0.016 eV [44]), acetic acid (–1.302 to –0.482 eV [45]), and acrylamide (–0.585 eV [46]) generally have substantially lower or even negative electron affinities. Judging from these EA values, it seems possible that gaseous peptides, even with zero net charge, can feature salt bridge structures, but direct experimental evidence for this has so far been demonstrated only for (RR + H)^+^ ions [47].

Furthermore, can we rationalize the observed linear correlation of CR energy with charge divided by the cube root of the number of atoms (Figure 2a)? Because ion volume should be roughly proportional to the number of atoms, the cube root of the number of atoms can be interpreted as the average value of the ion’s three dimensions. The linear correlation of CR energy with charge divided by the cube root of the number of atoms shown in Figure 2a is thus consistent with simple Coulomb interactions dominating cation-electron charge recombination in roughly spherical ion structures. From the fit in Figure 2a, adiabatic CR energies between 5.1 and 12.0 eV can be estimated for ubiquitin (M + nH)^n+^ ions with *n* = 5–13, which corresponds to ~1–3 meV or ~0.1–0.3 kJ/mol per vibrational degree of freedom (DOF, calculated as three times the number of atoms minus 6) as shown in Figure 2b. Nevertheless, this estimate for protein ion CR energies is far higher than that of 4–7 eV by Zubarev [48] who used a thermodynamic cycle that comprised apparent proton affinities of (M + nH)^n+^ ions [49].

However large or small, the CR energies may not be fully available for potential ion heating as part of it can be consumed by backbone cleavage. N–Cα bond dissociation energies between –0.92 and 1.63 eV were calculated for small radical peptide model systems [42], indicating that the excess energy in ECD can be substantially lower than the energy from charge recombination. Moreover, even the exoergic reactions with negative bond dissociation energies can be slowed by barriers, for which transition state energies between 0.02 and 0.99 eV were calculated [42]. Computing CR and dissociation energies for peptide and protein (M + nH)^(n-1)+•^ ions remains nonetheless challenging, not least because the exact charge locations and structures of most gaseous peptides and essentially all protein ions are unknown.

To illustrate the intricate interplay of charge location and structure in gaseous ions, three examples are given here. First, even for compounds as simple as para-aminobenzoic acid methyl ester, the preferred protonation site changes from the carbonyl oxygen to the amine nitrogen upon relatively small changes in chemical environment (i.e., by increasing the number of attached water molecules from 2 to 3 [50]). Translating this scenario to gaseous peptide or protein ions, even relatively small local structural changes such as sidechain reorientations, especially those involving hydrogen bonds, are likely to significantly affect the proton affinities of individual sites and, therefore, the CR energies. Second, ion mobility spectrometry (IMS) studies showed that simple crown ether binding can, depending on ion net charge, decrease or increase collision cross-sections of gaseous protein ions by more than 30% [51], indicating major structural changes caused by minor local binding events. Third, positioning of negative and positive charge on the N- and C-terminus of polyalanine peptides, respectively, stabilizes α-helical structures through favorable charge–helix dipole interactions, whereas polyalanine peptides with opposite charge locations adopt globular structures [52]. The above findings exemplify the complexity of gaseous protein ions structures [53], which is further increased by the possibility of kinetic trapping of less stable structures from electrospray ionization (ESI) [54].

McLuckey and coworkers have recently investigated the effect of recombination energy by comparing ECD spectra of small peptides to those from electron transfer dissociation (ETD), in which the recombination energy should be smaller than in ECD by the electron affinity of the reagent used, 0.6 eV for azobenzene and 1.7 eV for 1,3-dinitrobenzene [55]. Data interpretation was complicated by the fact that products from sequential electron transfer and H^•^ loss could not be distinguished from products from competitive proton transfer. However, the partitioning among the various ***c***, ***z*** ^•^ ion channels was remarkably similar in ECD and ETD for most of the model peptides studied, which would argue against any effect of charge recombination energy on peptide ion structure prior to backbone cleavage into ***c*** and ***z*** ^•^ ions.

**Figure 3:**
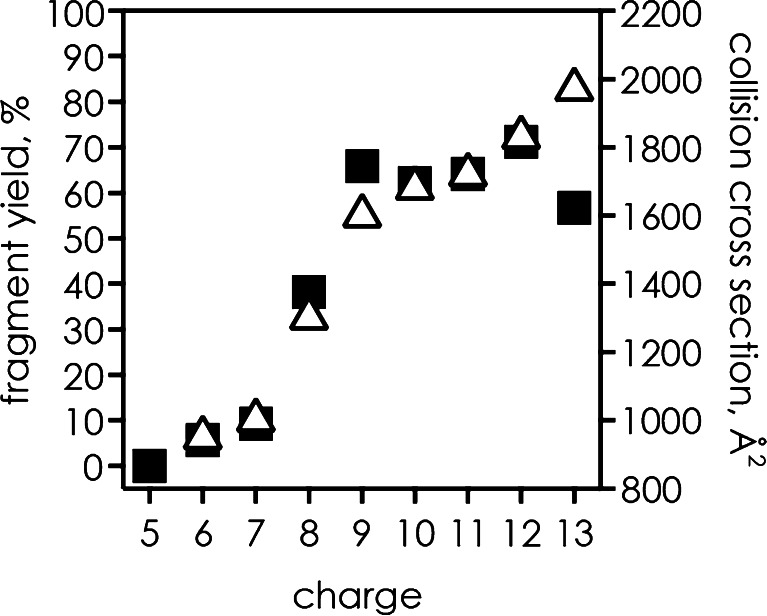
Yield of ***c***, ***z*** ^•^ fragments from ECD (*squares*, left axis) and collision cross-sections (*triangles*, right axis) of ubiquitin (M + nH)^n+^ ions at room temperature versus charge (data from references [9], [56])

The above discussion considered mostly peptides, but is there any experimental evidence for protein ion unfolding caused by ECD? The strong correlation of ***c*** and ***z*** ^•^ ion yields with collision cross-sections found for ubiquitin [9] (Figure 3) suggests that any excess energy deposited by electron capture does not significantly affect protein ion higher order structure on the timescale of the ECD experiment. This hypothesis is corroborated by data from ECD of different ubiquitin conformers of the same charge, separated by ion mobility, which showed significant differences in both overall and site-specific fragment ion abundances [17]. More specifically, any ubiquitin ion unfolding caused by electron capture must be far slower than the timescale for structural probing by ECD of typically several tenths of milliseconds, or the excess energy must be insufficient to cause significant unfolding, possibly due to fast radiative cooling by emission of infrared or higher-energy photons [57].

Radiative cooling by emission of infrared photons can be a complex process with rates that generally increase with increasing internal energy of the ions (i.e., hotter ions cool faster). For example, Al_4_^–^ clusters showed a decrease in radiation intensity from 40 eV/s at 1127°C to 1 eV/s at 227°C [58]. Nevertheless, Dunbar pointed out that “often it will be a reasonable approximation to think of the cooling as exponential” such that the internal energy decay of a population of superthermal ions can be described in terms of first-order cooling rate constants [59]. For small non-halogenated organic ions, infrared radiative cooling rate constants between 0.5 and 15 s^–1^ at energies between 0.3 and 4 eV have been measured [59], and those for singly protonated pentapeptide leucine enkephalin ions were deduced from experimental data as 30 s^–1^ at 165°C [60] and 7.5 ± 0.5 s^–1^ at ~25°C [61]. The internal energy of (M + H)^+^ ions of leucine enkephalin at 25°C is ~1 eV [62], which can be increased to ~3 eV (at which the cooling rate constant of 7.5 ± 0.5 s^–1^ was determined) [63] in tandem MS experiments to overcome the activation energy of 1.09 ± 0.06 eV [64] for dissociation into typical products from slow ion heating [65] within a few milliseconds [66]. From the fit in Figure 2a, CR energies of 2.90 and 5.09 eV can be estimated for electron attachment to (M + H)^+^ and (M + 2H)^2+^ ions of leucine enkephalin, respectively, which should be sufficient for dissociation into ***b*** and ***y*** fragments on a milliseconds timescale. Whereas the uncharged products from ECD of (M + H)^+^ ions cannot be directly detected, ECD of (M + 2H)^2+^ ions of leucine enkephalin in fact produced no ***c*** or ***z*** ^•^ but instead ***a***, ***b***, ***y*** and ***w*** ions [67]. It has been suggested that ***b*** ions form after loss of H^•^ from “hot” (M + 2H)^+•^ ions, the probability for which depends on peptide composition, structure, and internal energy [68]. Nevertheless, the formation of ***b*** ions in ECD of proteins and most peptides is rare, consistent with nanocalorimetry ECD experiments of partially hydrated (KYK + 2H)^2+^ ions, which gave both ***c*** and ***b*** ions, but ***c*** ion formation required only ~14% of the energy required for ***b*** ion formation [43].

**Figure 4:**
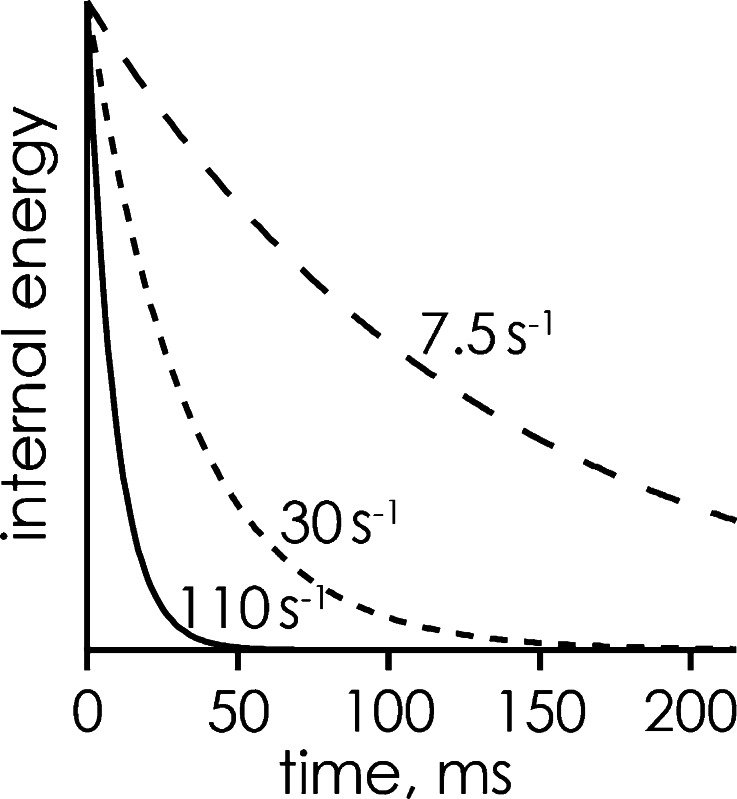
Internal energy versus time assuming exponential decay, calculated for the cooling rate constants indicated

The estimated CR energies of 2.90 and 5.09 eV for leucine enkephalin correspond to 13 and 22 meV deposited per DOF, which is about an order of magnitude higher than the estimated values of ~1–3 meV for ubiquitin ions (Figure 2b). Moreover, larger peptide and protein ions in the rapid energy-exchange (REX) limit should thermally equilibrate to the temperature of the surrounding vacuum system, typically room temperature, at rates that are the same as in the high pressure limit [69, 70]. The corresponding cooling rate constants should by far exceed the ~110 s^–1^ measured for singly protonated leucine enkephaline at 3.7 × 10^–4^ mbar and 25°C [60], such that cooling to room temperature of larger peptide and protein ions by emission of infrared photons should be possible within a few milliseconds (Figure 4).

Conversion of the CR energy, or parts of it, into IR radiation of course requires prior conversion into vibrational energy by intramolecular vibrational redistribution (IVR). It is generally agreed that electron capture involves higher-energy Rydberg states that undergo fast relaxation to lower energy Rydberg and then non-Rydberg (*n* < 3) electronic states [71], but whether this involves radiative or nonradiative relaxation by IVR, or both, is still unclear. Moreover, nothing is as yet known about radiative cooling of peptide or protein (M + nH)^n+•^ ions by emission of higher-energy photons, but the possibility that radiation from electronically excited states cools the radical ions formed by electron capture cannot generally be excluded [72]. Despite the gain in energy from charge recombination, the protein (M + nH)^(n-1)+•^ ions from electron capture are sufficiently stable for isolation and subsequent dissociation into ***c*** and ***z*** ^•^ (or ***c*** ^•^ and ***z***) fragments by IR laser radiation [9, 11, 20] at energies that are far lower than those required to dissociate (M + nH)^n+^ ions into ***b*** and ***y*** fragments. Possibly the radical (M + nH)^(n-1)+•^ ions formed by electron attachment into a Rydberg orbital with subsequent transfer to π* or σ* orbitals [34] also cool by emission of higher-energy photons from electronically excited states, which raises the “old” question if ECD can be described as a nonergodic process or if it is better described as a statistical process [1, 73, 74]. Anyhow, when cooling rates are significantly faster than the rates of unfolding, any excess energy from charge recombination should have little effect on the protein structures probed by ECD, but this may not be the case for small peptides. For example, Turecek and coworkers have calculated a temperature of ~580°C for a doubly charged pentapeptide after electron capture without radiative relaxation [75].

## Probing Protein Ion Structure

**Figure 5:**
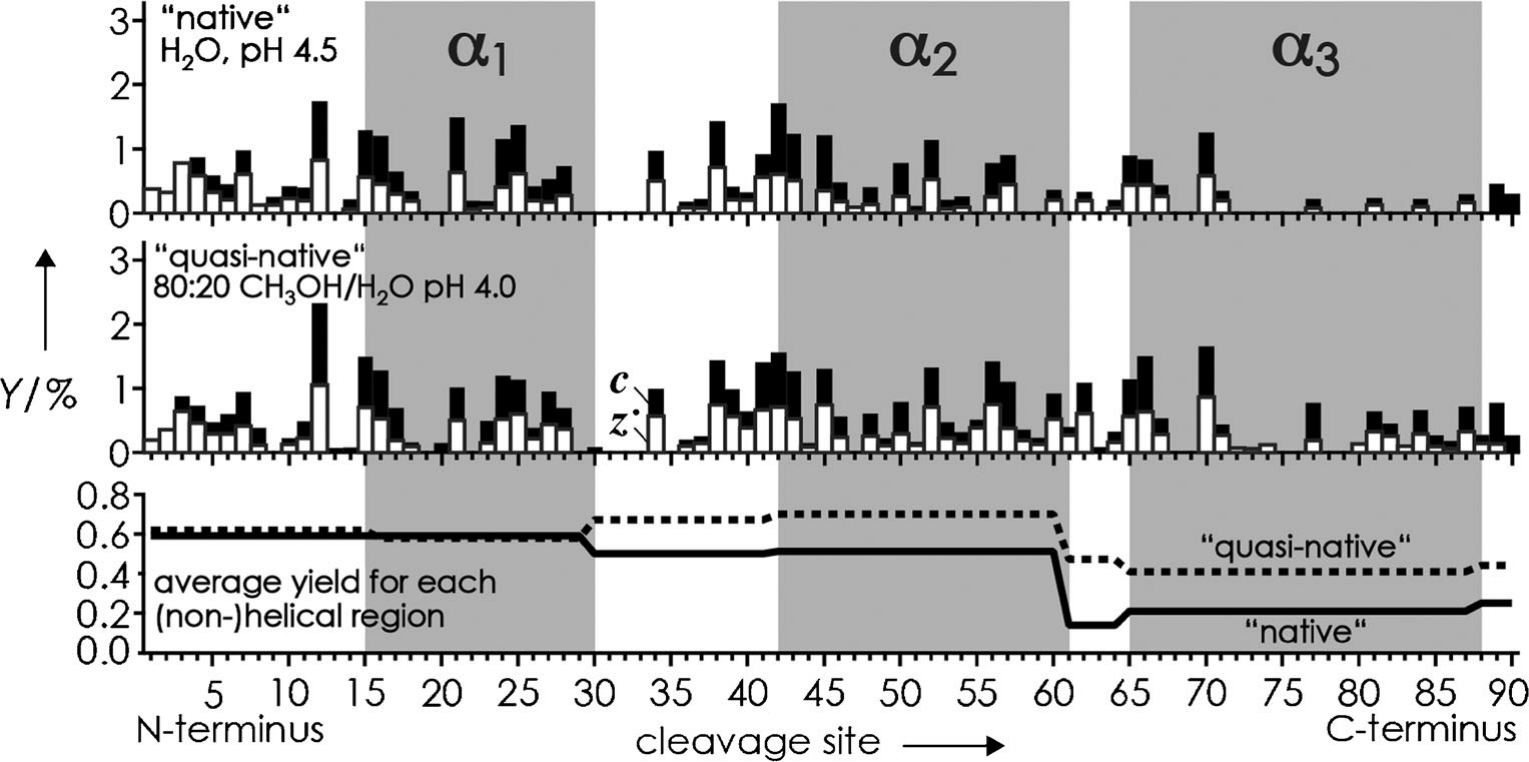
***c*** (*black bars*) and ***z*** ^•^ (*open bars*) yields versus cleavage site from ECD of KIX (M + 12H)^12+^ ions from ESI of “native” (*top*) and “quasi-native” (*center*) solutions, and average yield for each helical and non-helical region (*bottom*) for “native” (*solid line*) and “quasi-native” (*dashed line*) solutions; data from reference [19]

In a previous Critical Insight article, Hall and Robinson asked the important question “Do charge state signatures guarantee protein conformations?” and came to the conclusion that although experimental evidence “points to a consensus that the lowest charge states are the most compact,” none of the available techniques provides sufficient information for elucidation of gaseous protein structures [76]. Nevertheless, ion mobility spectrometry (IMS) studies showed that protein (M + nH)^n+^ ions with the same net charge but electrosprayed from different solutions can have considerably different collision cross-sections [56]. The first evidence that ECD can probe differences in protein structure that result from differences in solvent composition came from a study of the three-helix bundle protein KIX [19]. ECD of KIX (M + 12H)^12+^ ions electrosprayed from “native” and slightly denaturing (“quasi-native”) solutions gave total fragment ion yields^1^ of 37% and 49%, respectively, consistent with a higher extent of noncovalent bonding in ions from “native” solutions resulting in fewer separated fragments. Moreover, site-specific ***c***, ***z*** ^•^ ion yields revealed that denaturation unfolded the region comprising helices α_2_ and α_3_ (residues 30–91), whereas no overall effect was observed in the N-terminal region (Figure 5).

It is important to note that CAD differs markedly from ECD in this respect. For example, Clemmer and coworkers selected different conformers of ubiquitin (M + nH)^n+^ ions with *n* = 8–10 by ion mobility for subsequent dissociation by CAD, and found that the fragment mass spectra for different conformers of the same charge were identical within experimental error, which they attributed to rearrangement into structurally similar transition states prior to dissociation into ***b*** and ***y*** fragment ions [77]. Because CAD relies solely on vibrational excitation that incrementally increases an ion’s internal energy until dissociation occurs, it is reasonable that noncovalent bonds are broken before covalent bonds, unless the latter are weaker than the former.

Apart from higher order structure, the site-specific ***c***, ***z*** ^•^ ion yields from ECD are also affected by how positive charge is distributed and locally solvated within the protein ions [78], which gives rise to irregular (“jumpy”) features in plots of site-specific yield versus cleavage site, even when the protein ions are unfolded [9, 12, 19]. This is similar to steric and inductive effects of amino acid sidechains on hydrogen/deuterium exchange of protein backbone amides in solution, for which scalar correction factors have been established [79]. Delineating the effects of charge location and solvation in ECD is less straightforward, but it was recently shown that the “jumpy” features are due to distinct patterns termed charge site spectra [78] that can be retained over a wide range of precursor ion charge states for a given protein sequence. Finally, because ECD requires the presence of positive charge, a lack of ***c*** and ***z*** ^•^ fragments can also be observed in protein regions that lack any basic residues, or in protein regions that are bridged by disulfide bonds [20].

In any case, structural probing by ECD is indirect, meaning that it relies on observing separated ***c*** and ***z*** ^•^ fragments from protein backbone cleavage to reveal the *absence* of noncovalent bonding between them. Information about the number, types, or strengths of intramolecular interactions that prevent fragment ion separation cannot directly be inferred from a single ECD spectrum. In other words, structural probing by ECD provides an imprint of unfolded structure that shows which regions are unfolded, but not how and to what extent the other regions are folded. This is similar to footprinting techniques in solution in which folded regions can be shielded from, for example, hydroxyl radical attack, but with the major difference that in ECD, a single noncovalent interaction between two non-adjacent residues could prevent separation of all ***c*** and ***z*** ^•^ fragments from backbone cleavage in between these residues, no matter how compact that region is. Nevertheless, ECD provides valuable structural information, especially when data obtained under different experimental conditions are compared with each other (Figure 5), and when it is used to probe protein unfolding or folding [9, 10, 21, 80].

## Monitoring Protein Unfolding

Proteins can unfold for a number of different reasons, including solution pH, the presence of organic solvent or “denaturants” such as guanidinium hydrochloride, and elevated temperatures. As evidenced by ECD, the mere removal of solvent during ESI causes spontaneous unfolding of horse and tuna heart cytochromes *c* and ubiquitin, all of which have highly stable “native” folds in solution [21, 80]. The extent of unfolding in different regions of these proteins, however, was found to depend on the extent of stabilization by electrostatic interactions. Remarkably, the (M + 7H)^7+^ ions of the three-helix bundle protein KIX were sufficiently stabilized to fully preserve the native fold on a timescale of up to 4 s after transfer into the gas phase, and a gradual loss of higher order structure by sequential unraveling of the helices from their terminal ends was observed with increasing charge of the (M + nH)^n+^ ions [19]. Thus, ECD can be used to monitor unfolding in solution, or to probe structural changes that occur during the transition from solution into the gas phase, but distinguishing between these two possibilities is not straightforward when the solution structure is not known.

Regardless of whether a compact protein ion structure resembles the solution fold or not, its thermal unfolding in the gas phase can be monitored by ECD. For ubiquitin (M + 6H)^6+^ to (M + 9H)^9+^ ions that were thermally equilibrated by blackbody infrared radiation for at least 40 s, the yield of separated ***c***, ***z*** ^•^ fragments steadily increased with increasing temperature from 25 to 175°C [9]. Importantly, a van’t Hoff analysis of the ECD data revealed a three-state process for ubiquitin (M + nH)^n+^ ion unfolding in the gas phase, with ΔH values generally decreasing with increasing ion net charge. However, the ΔH values for *n* = 7 were similar to those for *n* = 6 at temperatures of up to 100°C, and similar to those for *n* = 8 at higher temperatures, showing a stepwise decrease in ΔH from ~30 to ~10 kJ/mol, in contrast to the gradual decrease of ΔH with decreasing pH for ubiquitin unfolding in solution [81]. Protein ions can also be unfolded by collisional activation prior to structural probing by ECD [80] or ETD [82], and although in such experiments carried out under multiple low-energy collisions it is usually not possible to determine ion temperatures and derive thermodynamic information, they can be used to establish the order of stability for different regions of a gaseous protein [21].

**Figure 6:**
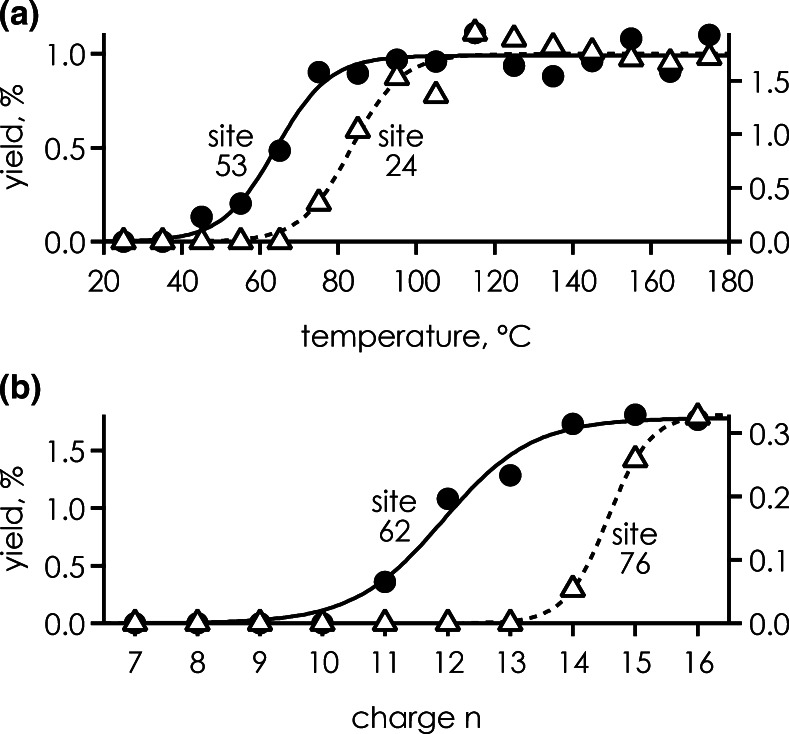
Site-specific yield of ***c*** and complementary ***z*** ^•^ fragments from ECD of (**a**) ubiquitin (M + 7H)^7+^ ions versus ion temperature for sites 24 (*right axis*) and 53 (*left axis*), data from reference [9], and (**b**) KIX (M + nH)^n+^ ions versus charge n for sites 62 (*left axis*) and 76 (*right axis*), data from reference [19]; *lines* represent sigmoidal fit functions

As discussed above, the site-specific fragment ion yields in ECD of protein (M + nH)^n+^ ions are not only affected by higher order structure but also by how positive charge is distributed and locally solvated within the protein ions studied (i.e., some variation in yield is found among all cleavage sites even in ECD of unfolded ions). Nevertheless, the site-specific ***c*** and ***z*** ^•^ ion yields generally showed sigmoidal behavior for (M + nH)^n+^ ion unfolding by temperature or charge *n* (Figure 6), from which melting temperatures [9] or transition charge values [19] can be derived, respectively. Monitoring the ***c*** and ***z*** ^•^ ion yields indicated different melting temperatures for different sites in the thermal unfolding of ubiquitin (M + 7H)^7+^ ions [e.g., 83 ± 2°C for site 53 and 64 ± 2°C for site 24 (Figure 6a)]. However, because ***z*** ^•^ ions can undergo secondary dissociation at elevated temperatures (Figure 1a), melting curves derived from only ***c*** instead of ***c*** and ***z*** ^•^ ions are more reliable in thermal unfolding experiments [9]. Thermal dissociation of ***z*** ^•^ ions should not be an issue in experiments in which protein ion unfolding is monitored by measuring site-specific ***c*** and ***z*** ^•^ ion yields with increasing (M + nH)^n+^ charge at room temperature (Figure 1b). The corresponding data also show sigmoidal transitions, as illustrated in Figure 6b for KIX (M + nH)^n+^ ions at sites 62 and 76. The scatter in transition charge values for adjacent sites can be substantial [19], which can be attributed to the loss of both local and global interactions. To eliminate the effect of interactions between adjacent residues and characterize the unfolding of protein higher order structure, integrated yields can be used for the determination of transition charge values for specific regions of the protein [19].

## Monitoring Protein Folding

A major drawback of structural probing by ECD is that a single spectrum does not reveal how much structure is retained in regions framed by two residues that are still held together by noncovalent bonding. This means that if gaseous protein ions were to fold by first associating their N- and C-terminal residues, all fragment ions would disappear simultaneously, no matter how compact or extended the structure were of the residues in between. However, for the proteins studied so far, horse and tuna heart cytochromes *c* and ubiquitin, such behavior has not been observed. Instead, regions in which the site-specific yield of ECD fragments decreased with increasing time allowed for folding (indicating the formation of noncovalent interactions between the complementary ***c*** and ***z*** ^•^ fragments from backbone cleavage in that region) were generally separated by regions in which the yield did not change (no new interactions formed), decreased to a lesser extent (fewer interactions formed), or even increased (loss of interactions) [9, 80]. Moreover, for these proteins, folding in the gas phase is not only slower than folding in solution by several orders of magnitude [10, 12, 80, 83, 84], it is also dominated by nearest neighbor interactions instead of global structural changes, and driven by electrostatic instead of hydrophobic interactions [80].

## Experimental Conditions for ECD

An important issue to be considered in ECD experiments for structural probing, as well as for sequencing applications, is secondary dissociation as a result of secondary (or multiple) electron capture. Because the cross-section for electron capture generally increases with the square of the net positive charge of an ion [2], the probability for secondary electron capture is generally higher for larger fragment ions that typically carry more charge than smaller fragments. Thus, under conditions of multiple electron capture, larger ***c*** and ***z*** ^•^ fragments are preferentially depleted, and only smaller fragments from backbone cleavage close to the protein termini are detected, which produces ECD spectra that can mistakenly be interpreted as reflecting noncovalent bonding in the center region of the protein. Such spectra are characterized by missing complementary ions, that is, only small ***c*** and ***z*** ^•^ fragments from cleavage close to the N and C terminus are detected, respectively. An example of such a spectrum can be found in Figure 1 of reference [20], and spectra recorded under conditions of single electron capture that show approximately equal yields of ***c*** and ***z*** ^•^ fragments at each cleavage site can be found in Figure 2 of reference [19].

## Open Questions and Future Challenges

ECD of proteins does not provide direct structural information, but the observation of ***c*** and ***z*** ^•^ fragments immediately reveals the absence of noncovalent bonding between them. Moreover, when changes in ECD spectra are followed as the temperature or charge of the protein ions under study is increased, or time is allowed for folding, site-specific data on the structural changes involved can be obtained. A major challenge for comprehensive structural studies is the analysis of complex ECD spectra. Although automated algorithms for spectral interpretation that can correctly assign and quantify the majority (~90%) of signals in an ECD spectrum are available, additional manual interpretation is, in our experience, absolutely essential. Regarding the mechanism of protein backbone bond cleavage into ***c*** and complementary ***z*** ^•^ ions and the implications for structural probing, it remains to be seen if there are lower mass and higher charge limits within which the energy gained by electron capture does not cause significant unfolding, and the role of electronically excited states in radiative cooling needs to be addressed. Finally, because charge locations in peptide and protein ions from ESI are critical to the development of theoretical models of the ECD process, a better understanding of the electrospray process and the structural changes involved is vitally important.

## Footnotes

1 ECD fragment ion yields are calculated as percentage values relative to all ECD products, considering that backbone dissociation of a parent ion gives a pair of complementary ***c*** and ***z*** ^•^ or a pair of ***a*** ^•^ and ***y*** ions, 100% = 0.5[***c***]. + 0.5[***a*** ^•^] + 0.5[***z*** ^•^] + 0.5[***y***] + [other products], in which other products are reduced molecular ions and products from loss of small neutral species from the latter. In cases where ***a*** ^•^ and ***y*** ions are of low abundance with correspondingly small signal-to-noise ratios, they can be excluded from the analysis.
